# Only one patient out of five achieves symmetrical knee function 6 months after primary anterior cruciate ligament reconstruction

**DOI:** 10.1007/s00167-019-05396-4

**Published:** 2019-02-18

**Authors:** Riccardo Cristiani, Christina Mikkelsen, Magnus Forssblad, Björn Engström, Anders Stålman

**Affiliations:** 1 Department of Molecular Medicine and Surgery, Stockholm Sports Trauma Research Center, Karolinska Institutet, Stockholm, Sweden; 2 Capio Artro Clinic, FIFA Medical Centre of Excellence, Sophiahemmet Hospital, Valhallavägen 91, 11486 Stockholm, Sweden

**Keywords:** Anterior cruciate ligament, ACL, Reconstruction, Quadriceps strength, Single leg hop test, Limb symmetry index, LSI, Rehabilitation, Knee function, Sports

## Abstract

**Purpose:**

To assess the percentage of patients achieving symmetrical knee function 6 months after primary anterior cruciate ligament (ACL) reconstruction (ACLR) and to identify factors affecting its achievement, in a large cohort.

**Methods:**

Data were extracted from our clinic database. Patients who underwent primary ACLR from 2000 to 2015 and were assessed with the isokinetic quadriceps and hamstring muscles strength tests and single-leg-hop test at the 6-month follow-up were included in the study. Demographic data, information on the graft used, cartilage injuries and concomitant meniscal surgery were reviewed. Patients who reached a limb symmetry index (LSI) of ≥ 90% in all three tests were considered to have achieved symmetrical knee function. A multivariate logistic regression analysis was used to determine whether patient age, gender, time from injury to surgery, pre-injury Tegner activity level, graft type, cartilage injury and the presence of medial meniscus (MM) or lateral meniscus (LM) resection or repair were factors associated with the achievement of symmetrical knee function 6 months after primary ACLR.

**Results:**

A total of 4093 patients (54.3% males) with a mean age of 28.3 ± 10.7 years were included. Data from all three tests were available for 3541 patients. The proportion of patients that achieved a LSI of ≥ 90% was 35.7%, 47.3% and 67.9% for isokinetic quadriceps muscle strength, hamstring muscles strength and the single-leg-hop test, respectively. A total of 693 patients (19.6%) achieved symmetrical knee function, reaching a LSI of ≥ 90% in all three tests. Older age (≥ 30 years) (OR, 0.50; 95% CI 0.41–0.61; *P* < 0.001), MM resection (OR, 0.75; 95% CI 0.57–0.98; *P* = 0.03) and MM repair (OR, 0.63; 95% CI 0.40–0.98; *P* = 0.04) reduced the odds, whereas the use of hamstring tendon (HT) autograft (OR, 2.28; 95% CI 1.51–3.45; *P* < 0.001) over bone–patellar tendon–bone (BPTB) autograft increased the odds of achieving symmetrical knee function.

**Conclusion:**

Only 19.6% of the patients achieved symmetrical knee function 6 months after primary ACLR. Age ≥ 30 years, MM resection and MM repair reduced the chance, whereas the use of HT autograft over BPTB autograft increased the chance of achieving symmetrical knee function 6 months after primary ACLR. This study shows that most of the patients are yet to regain symmetrical knee function 6 months after primary ACLR and, moreover, it identifies several factors affecting its achievement in a large cohort. The results of this study should be used to counsel patients about their expected functional recovery and to optimize rehabilitation and maximize knee function after ACLR.

**Level of evidence:**

III.

## Introduction

An anterior cruciate ligament (ACL) tear is a serious knee injury that can prevent patients from participating in both daily living and physical activities [1, 21]. ACL reconstruction (ACLR) is common among patients who want to return to sport (RTS) [3, 18]. RTS is often advocated 6 months after ACLR [4]. However, this timeframe has recently been questioned, as the risk of sustaining an ACL re-injury is highest during the early period (6–12 months) of RTS [8, 17]. Post-surgical time alone is not sufficient to determine readiness for RTS [8]. Functional tests, such as isokinetic muscle strength tests and the single-leg-hop test, are regarded as key factors prior to RTS [8, 34, 37]. The results of these tests are most frequently reported through the limb symmetry index (LSI) [34, 35]. The LSI is a measurement of the strength/function of the operated limb in relation to the healthy limb. The rationale is to ensure that the injured leg reaches an acceptable level of strength and function when returning to sport or strenuous activities. A LSI of ≥ 90% in these tests is the threshold value that should be reached for a successful and safe RTS [9, 13, 21, 31, 34, 43]. Muscular asymmetries not only affect sport performance, they are also associated with a greater risk of ACL graft rupture and knee re-injuries after RTS [8, 17]. Single-legged hop tests are able to discriminate between those individuals who return to their previous activity level from those who do not after ACLR [1]. Patients with symmetrical isokinetic muscle strength and hop performance 6 months after ACLR have superior knee function and higher activity levels at the midterm follow-up [31]. Therefore, rehabilitation should assist in restoring limb-to-limb symmetry.

To date, no previous studies have assessed the rate of patients achieving symmetrical knee function, measured as symmetry in isokinetic quadriceps and hamstring muscles strength and single-leg-hop test performance, 6 months after primary ACLR, in a large cohort. In addition, a detailed analysis of patients’ factors affecting the achievement of symmetrical knee function has not previously been presented. The heterogeneity of patients’ preoperative and intraoperative variables makes it difficult to predict which patients will be able to achieve symmetrical knee function 6 months after ACLR. However, this information would be very valuable for clinicians and physiotherapists to counsel patients about their expectations regarding functional recovery and to optimize rehabilitation and maximize knee function after ACLR.

The purpose of this study was to assess the percentage of patients achieving symmetrical knee function 6 months after primary ACLR and to identify factors affecting its achievement, in a large cohort. It was hypothesized that age, gender, graft choice, concomitant meniscal surgery and cartilage injuries would affect the chance of achieving symmetrical knee function at the 6-month follow-up.

## Materials and methods

Data were extracted from our clinic database. A total of 4341 patients who underwent primary ACLR from 2000 to 2015 and were assessed at the 6-month follow-up with the isokinetic quadriceps and hamstring muscles strength tests and the single-leg-hop test were identified. All the included patients had no concomitant ligament injuries. Patients who had contralateral ACL injuries or reconstructions (*n* = 248) were excluded.

### Surgical technique and rehabilitation

All the patients were operated on using a single-bundle autologous hamstring tendon (HT) or bone–patellar tendon–bone (BPTB) technique. For the ACLRs performed with HT graft, the semitendinosus tendon was primarily harvested and prepared as a triple or quadruple graft. If the length or the diameter of the graft was considered insufficient (< 8 mm), the gracilis tendon was additionally harvested and combined with the semitendinosus graft. The BPTB graft was harvested as the central third of the patellar tendon with two bone blocks. Both grafts were routinely fixed using an Endobutton fixation device (Smith & Nephew, Andover, Mass, USA) on the femoral side and Ethibond no. 2 sutures (Ethicon, Sommerville, NJ) tied over an AO bicortical screw with a washer as a post or using an interference screw on the tibial side. Meniscal repair was performed, for both the medial meniscus (MM) and lateral meniscus (LM), with an arthroscopic all-inside technique using a Fast-Fix suture anchor device (Smith and Nephew, Andover, Mass, USA) or an inside-out technique for tears located in the dorsal and middle portion of the meniscus. An outside-in technique was used for tears located in the anterior portion of the meniscus. Both inside-out and outside-in meniscal repair techniques were performed using PDS 0 (Ethicon, Sommerville, NJ). All the patients followed a standardized postoperative rehabilitation protocol. In the event of an isolated ACLR or an ACLR with simultaneous meniscal resection, full weight bearing and full range of motion were encouraged as tolerated. If a meniscal repair was performed, patients wore a hinged knee brace for 6 weeks. Flexion was limited from 0° to 30° for the first 2 weeks, from 0° to 60° for the third and fourth weeks, and from 0° to 90° for the fifth and sixth weeks after surgery. Starting from the seventh week, the knee brace was discontinued and progressive weight bearing was allowed. The early rehabilitation phase focused on regaining range of motion, reduction of swelling and gait correction. The rehabilitation protocol included joint and muscle flexibility exercises, balance/coordination training and strength training focusing primarily on the thigh muscles. For all patients, quadriceps strengthening was restricted to closed kinetic chain exercises during the first 3 months. On the basis of muscle strength, functional hop performance and type of sport, the patients were allowed to RTS 6 months postoperatively at the earliest.

### Outcome

All the patients were evaluated at our outpatient clinic 6 months postoperatively, using a standardized protocol. Isokinetic concentric quadriceps and hamstring muscles strength was measured bilaterally at 90°/s using the Biodex System 3 (Biodex Medical Systems, Shirley, New York, USA) [6]. The test was performed in a range of motion between 90° and 10° of knee flexion, always starting with the contralateral uninjured knee. Prior to the test, the patients warmed up using a stationary cycling ergometer at low resistance for 10 min. Patients were given a verbal description of the test and two–three practical trials were allowed before testing. Each patient performed five maximal quadriceps and hamstring muscles contractions with each leg. Patients were verbally encouraged during the test. The peak quadriceps and hamstring torque values (highest achieved values) were registered.

The single-leg-hop test was used to assess functional hop performance [25, 27]. The test was performed with the patient standing on one leg and being instructed to jump straight ahead as far as possible and land on the same leg. The test was considered successful if the landing was stable. If the patient landed with an early touchdown of the contralateral limb, had a loss of balance or took additional hops after landing, the hop was repeated. Patients were initially given a verbal description of the test and they were allowed to perform as many practical trials as they wanted, until they felt confident about the test. Three trials were performed for each leg, always starting with the contralateral uninjured leg [2]. Patients were given as much time as they wanted between the trials to minimize fatigue. The best trial for each leg was registered. Patients who did not want to undertake the test, who did not feel confident enough about the test or who did not undertake jumping exercises during the rehabilitation were not tested.

All three tests (isokinetic quadriceps and hamstring muscles strength tests and single-leg-hop test) were conducted by experienced sports medicine physiotherapists who had undergone extensive training in these testing procedures. The limb symmetry indexes (LSIs) of the peak quadriceps and hamstring muscles torque and single-leg-hop test were calculated as [involved limb/uninvolved limb × 100] for each test. The achievement of a symmetrical isokinetic muscle strength or single leg hop test performance was defined as performing at least 90% of the contralateral leg (LSI ≥ 90%) [9, 24, 34]. Patients who reached a LSI of ≥ 90% in all three tests were considered to have achieved symmetrical knee function.

### Data sources

Demographic data (age and gender), information about the time from injury to surgery, pre-injury Tegner activity level [32], graft type, meniscus surgery and the presence of cartilage injuries were collected in our local database. Meniscus surgery was classified as follows: no meniscus surgery, meniscus resection, or meniscus repair for both medial and lateral meniscus. The results of the isokinetic quadriceps and hamstring muscles strength tests and the single-leg-hop test 6 months following ACLR were reviewed.

Ethical permission for this study was obtained from the regional ethics committee, Karolinska Institutet (Diarienumber 2016/1613-31/32).

### Statistical analysis

The Statistical Package for Social Sciences, SPSS (Version 25.0, IBM Corp., Armonk, New York, USA) was used for the statistics. All variables were summarized with standard descriptive statistics such as mean, standard deviations, or frequency. Symmetrical isokinetic quadriceps or hamstring muscles strength and single-leg-hop test performance were defined as achieving a LSI of ≥ 90% (“Yes” vs. “No”) in each test and were used as outcome measure. The relationship between the three outcome measures (quadriceps muscle strength, hamstring muscles strength and single-leg-hop test performance) was investigated using the phi-coefficient. Three separate multivariate logistic regression analysis—isokinetic quadriceps muscle strength, hamstring muscles strength, single-leg-hop test performance—were performed with age, gender (female vs. male), time from injury to surgery (delayed >3 months vs. not delayed ≤ 3 months), pre-injury Tegner activity level (high ≥ 6 vs. low < 6), graft (HT vs. BPTB autograft), medial meniscus resection, medial meniscus repair, lateral meniscus resection, lateral meniscus repair, and cartilage injury as independent variables and the achievement of symmetrical (LSI ≥ 90%) isokinetic quadriceps muscle strength, hamstring muscles strength and single-leg-hop test performance as dependent variables. An additional multivariate logistic regression analysis was performed with the same independent variables and the achievement of a LSI of ≥ 90% in all three tests (symmetrical knee function) as the dependent variable. Age was dichotomized into classes close to the median (≥ 30 years vs. < 30 years). The relationships were expressed as odds ratios (OR) with 95% confidence intervals (CI). The level of significance in all analysis was 5% (two-tailed).

Provided a significance level of 5%, a power of 85%, and a sample size of more than 1000 patients, even a very weak relationship of less than 0.10 (phi coefficient), which corresponds to an effect size of less than 0.10 according to Cohen, would be detected.

## Results

A total of 4,093 patients, with 6-month isokinetic quadriceps and hamstring muscles strength tests data, formed the study cohort. Of these, data for the single-leg-hop test were available for 3541 patients. Patient characteristics are summarized in Table 1.

**Table 1:** Patient characteristics (*n* = 4093 Patients)

		Quadriceps strength ratio		Hamstring strength ratio		Single-leg-hop test ratio^a^	
		Mean ± SD	LSI ≥ 90%	Mean ± SD	LSI ≥ 90%	Mean ± SD	LSI ≥ 90%
Preoperative factors							
Age at surgery, years, mean ± SD	28.3 ± 10.7						
Aged younger than 30 years	20.7 ± 4.7; 2408 (58.8)	86.5 ± 14.8	992 (41.2)	91.0 ± 22.4	1194 (49.6)	94.2 ± 10.8	1579 (72.7)
Aged 30 years or older	39.1 ± 6.9; 1685 (41.2)	81.5 ± 16.4	445 (26.4)	88.6 ± 15.4	741 (44.0)	90.2 ± 15.0	828 (60.4)
Gender							
Male	2223 (54.3)	84.8 ± 15.9	804 (36.2)	90.7 ± 22.8	1078 (48.5)	93.4 ± 12.2	1377 (71.5)
Female	1870 (45.7)	84.0 ± 15.4	633 (33.8)	89.2 ± 15.6	856 (45.8)	91.7 ±13.5	1029 (63.7)
Time from injury to surgery, months, mean ± SD	16.9 ± 29.8						
≤ 3 months	646 (15.8)	84.0 ± 15.2	234 (36.2)	91.1 ± 12.6	342 (52.9)	94.4 ± 10.0	439 (75.8)
> 3 months	3447 (84.2)	84.6 ± 15.6	1202 (34.9)	89.6 ± 16.0	1593 (46.2)	92.3 ± 13.3	1967 (66.4)
Pre-injury Tegner activity level, median (range)	7 (1–10)						
High, ≥ 6	3618 (88.4)	84.7 ± 15.2	1286 (35.6)	90.0 ± 15.2	1733 (47.9)	93.1 ± 12.6	2208 (69.7)
Low, < 6	475 (11.6)	82.7 ± 17.8	150 (31.6)	88.5 ± 17.8	206 (43.4)	88.8 ± 13.9	199 (53.3)
Intraoperative factors							
Graft type							
HT autograft	3788 (92.5)	85.1 ± 15.2	1387 (36.6)	89.3 ± 15.3	1726 (45.6)	92.8 ± 12.7	2267 (68.8)
BPTB autograft	305 (7.5)	76.1 ± 17.1	49 (16.1)	96.6 ±16.8	209 (68.5)	89.9 ± 13.4	141 (57.5)
No meniscus surgery	2577 (63.0)	84.7 ± 15.9	906 (35.2)	89.9 ± 15.1	1210 (47.0)	93.0 ± 12.8	1502 (58.3)
Medial meniscus surgery							
Resection	578 (14.1)	84.0 ± 14.8	178 (30.8)	88.7 ± 15.3	258 (44.6)	90.3 ± 13.9	298 (60.9)
Repair	194 (4.7)	81.9 ± 15.6	53 (27.3)	89.7 ± 13.9	92 (47.4)	91.6 ± 11.4	105 (61.4)
Lateral meniscus surgery							
Resection	594 (14.5)	84.2 ± 13.9	208 (35.0)	89.5 ± 13.9	281 (47.3)	93.1 ± 10.7	364 (61.3)
Repair	150 (3.7)	82.3 ± 14.4	43 (28.7)	92.6 ± 20.6	71 (47.3)	92.8 ± 16.6	80 (62.5)
Cartilage injury							
Yes	738 (18.3)	82.3 ± 16.6	222 (30.1)	88.3 ± 14.9	308 (41.7)	90.5 ± 14.4	362 (60.4)
No	3355 (81.7)	84.9 ± 15.3	1214 (36.2)	90.2 ± 15.7	1631 (48.6)	93.1 ± 12.4	2044 (69.5)

Data are reported as n (%), unless otherwise indicated

*HT* hamstring tendon, *BPTB* bone–patellar tendon–bone, *LSI* limb symmetry index, *SD* standard deviation

^a^Data available for 3541 patients

The relationship between the three outcome measures is shown in Table 2.

**Table 2:** Relationships between the outcome measures investigated with the Phi coefficient

Outcome variable	Quadriceps strength LSI ≥ 90%	Hamstring strength LSI ≥ 90%	Single-leg-hop test LSI ≥ 90%
Quadriceps strength LSI ≥ 90%	–	0.22^a^	0.26^a^
Hamstring strength LSI ≥ 90%		–	0.11^b^
Single-leg-hop test LSI ≥ 90%			

*LSI* limb symmetry index

^a^Weak positive correlation (Phi coefficient between 0.20 and 0.29)

^b^No or negligible correlation (Phi coefficient between 0.01 and 0.19)

### Isokinetic quadriceps muscle strength

A total of 1463 patients (35.7%) in the studied cohort achieved symmetrical isokinetic quadriceps muscle strength. Age ≥ 30 years, odds ratio [OR] 0.47; 95% confidence interval [CI] 0.41–0.55; *P* < 0.001), female gender (OR, 0.80; 95% CI 0.70–0.91; *P* = 0.001), MM repair (OR, 0.60; 95% CI 0.43–0.84; *P* = 0.003) and LM repair (OR, 0.61; 95% CI 0.42–0.89; *P* = 0.01) reduced the odds, whereas the use of HT autograft (OR, 3.41; 95% CI 2.48–4.69; *P* < 0.001) over BPTB autograft increased the odds of achieving symmetrical quadriceps muscle strength. No correlation was found between the achievement of a LSI of ≥ 90% for quadriceps muscle strength and the time from injury to surgery, pre-injury Tegner activity level, cartilage injury and MM or LM resection (Table 3).

**Table 3:** Factors associated with the achievement of symmetrical (LSI ≥ 90%) *isokinetic quadriceps muscle strength* after ACL reconstruction in multivariate logistic regression analysis (*n* = 4093 Patients)

Factor	Regression coefficient (*ß*)	SE	O.R. (95% CI)	*P* value
Age ≥ 30 years	− 0.73	0.07	0.47 (0.41–0.55)	< 0.001*
Female gender	− 0.22	0.06	0.80 (0.70–0.91)	0.001*
Delayed (> 3 months) ACLR	0.05	0.09	1.05 (0.87–1.26)	ns
Pre-injury Tegner activity level ≥ 6	− 0.06	0.11	0.93 (0.75–1.16)	ns
HT autograft	1.22	0.16	3.41 (2.48–4.69)	< 0.001*
MM resection	− 0.16	0.10	0.84 (0.69–1.02)	ns
MM repair	− 0.49	0.16	0.60 (0.43–0.84)	0.003*
LM resection	− 0.05	0.09	0.94 (0.78–1.14)	ns
LM repair	− 0.48	0.18	0.61 (0.42–0.89)	0.01*
Cartilage injury	− 0.09	0.09	0.91 (0.76–1.09)	ns

*LSI* limb symmetry index, *ACL* anterior cruciate ligament, *ACLR* anterior cruciate ligament reconstruction, *HT* hamstring tendon, *MM* medial meniscus, *LM* lateral meniscus, *SE* standard error, *OR* odds ratio, *CI* confidence interval

*Statistically significant. *P* value < 0.05

### Isokinetic hamstring muscles strength

The total number of patients who achieved symmetrical isokinetic hamstring muscles strength was 1,935 (47.3%). Age ≥ 30 years (OR, 0.85; 95% CI 0.74–0.97; *P* = 0.02), delayed (> 3 months) ACLR (OR, 0.81; 95% CI 0.68–0.97; *P* = 0.02), HT autograft (OR, 0.40; 95% CI 0.31–0.51; *P* < 0.001) and cartilage injury (OR, 0.82; 95% CI 0.69–0.97; *P* = 0.02) reduced the odds of achieving symmetrical hamstring muscles strength. No other preoperative or intraoperative patient factors were found to be associated with the achievement of a LSI of ≥ 90% for hamstring muscles strength (Table 4).

**Table 4:** Factors associated with the achievement of symmetrical (LSI ≥ 90%) isokinetic hamstring muscles strength after ACL reconstruction in multivariate logistic regression analysis (*n* = 4093 Patients)

Factor	Regression coefficient (*ß*)	SE	OR (95% CI)	*P* value
Age ≥ 30 years	− 0.15	0.06	0.85 (0.74–0.97)	0.02*
Female gender	− 0.08	0.06	0.91 (0.80–1.04)	ns
Delayed (> 3 months) ACLR	− 0.20	0.08	0.81 (0.68–0.97)	0.02*
Pre-injury Tegner activity level ≥ 6	0.11	0.10	1.12 (0.91–1.37)	ns
HT autograft	− 0.90	0.12	0.40 (0.31–0.51)	< 0.001*
MM resection	− 0.04	0.09	0.95 (0.79–1.14)	ns
MM repair	0.02	0.15	1.02 (0.76–1.37)	ns
LM resection	− 0.02	0.09	0.97 (0.81–1.16)	ns
LM repair	− 0.03	0.17	0.96 (0.69–1.34)	ns
Cartilage injury	− 0.19	0.08	0.82 (0.69–0.97)	0.02*

*LSI* limb symmetry index, *ACL* anterior cruciate ligament, *ACLR* anterior cruciate ligament reconstruction, *HT* hamstring tendon, *MM* medial meniscus, *LM* lateral meniscus, *SE* standard error, *OR* odds ratio, *CI* confidence interval

*Statistically significant. *P* value < 0.05

### Single-leg-hop test performance

The patients who achieved a symmetrical performance in the operated limb in relation to the healthy limb for the single-leg-hop test were 2,405 (67.9%). Age ≥ 30 years (OR, 0.60; 95% CI, 0.51–0.70; *P* < 0.001), female gender (OR, 0.64; 95% CI 0.55–0.75; *P* < 0.001), delayed (> 3 months) ACLR (OR, 0.70; 95% CI 0.57–0.87; *P* < 0.001), MM resection (OR, 0.73; 95% CI 0.60–0.90; *P* = 0.003), MM repair (OR, 0.71; 95% CI 0.51–0.99; *P* = 0.04) and cartilage injury (OR, 0.78; 95% CI 0.65–0.95; *P* = 0.01) reduced the odds, whereas high pre-injury Tegner activity level (≥ 6) (OR, 1.59; 95% CI 1.26–1.99; *P* < 0.001) and the use of HT autograft (OR 1.95; 95% CI 1.48–2.56; *P* < 0.001) over BPTB autograft increased the odds of achieving a symmetrical single-leg-hop test performance. No correlation was found between the achievement of a LSI of ≥ 90% for the single-leg-hop test and LM resection or repair (Table 5).

**Table 5:** Factors associated with the achievement of symmetrical (LSI ≥ 90%) single leg hop test performance after ACL reconstruction in multivariate logistic regression analysis (*n* = 3541 Patients)

Factor	Regression coefficient (*ß*)	SE	OR (95% CI)	*P* value
Age ≥ 30 years	− 0.51	0.07	0.60 (0.51–0.70)	< 0.001*
Female gender	− 0.43	0.07	0.64 (0.55–0.75)	< 0.001*
Delayed (> 3 months) ACLR	− 0.34	0.10	0.70 (0.57–0.87)	< 0.001*
Pre-injury Tegner activity level ≥ 6	0.46	0.11	1.59 (1.26–1.99)	< 0.001*
HT autograft	0.66	0.13	1.95 (1.48–2.56)	< 0.001*
MM resection	− 0.30	0.10	0.73 (0.60–0.90)	0.003*
MM repair	− 0.34	0.17	0.71 (0.51–0.99)	0.04*
LM resection	0.10	0.10	1.11 (0.90–1.38)	ns
LM repair	− 0.38	0.19	0.68 (0.47–1.00)	ns
Cartilage injury	− 0.24	0.09	0.78 (0.65–0.95)	0.01*

*LSI* limb symmetry index, *ACL* anterior cruciate ligament, *ACLR* anterior cruciate ligament reconstruction, *HT* hamstring tendon, *MM* medial meniscus, *LM* lateral meniscus, *SE* standard error, *OR* odds ratio, *CI* confidence interval

*Statistically significant. *P* value < 0.05

### Symmetrical knee function

A total of 693 patients (19.6%) achieved symmetrical knee function, reaching a LSI of ≥ 90% in all three tests (isokinetic quadriceps and hamstring muscles strength tests and single-leg-hop test). Age ≥ 30 years (OR, 0.50; 95% CI 0.41–0.61; *P* < 0.001), MM resection (OR, 0.75; 95% CI, 0.57–0.98; *P* = 0.03) and MM repair (OR, 0.63; 95% CI 0.40–0.98; *P* = 0.04) reduced the odds, whereas the use of HT autograft (OR, 2.28; 95% CI 1.51–3.45; *P* < 0.001) over BPTB autograft increased the odds of achieving symmetrical knee function (Table 6).

**Table 6:** Factors associated with the achievement of *symmetrical knee function* (LSI ≥ 90% for isokinetic quadriceps and hamstring muscles strength and single-leg-hop test performance) after ACL reconstruction in multivariate logistic regression analysis (*n* = 3541 Patients)

Factor	Regression coefficient (ß)	SE	OR (95% CI)	*P* value
Age ≥ 30 years	− 0.68	0.09	0.50 (0.41–0.61)	< 0.001*
Female gender	− 0.14	0.08	0.86 (0.72–1.02)	ns
Delayed (> 3 months) ACLR	− 0.11	0.11	0.89 (0.71–1.11)	ns
Pre-injury Tegner activity level ≥ 6	0.04	0.15	1.04 (0.77–1.40)	ns
HT autograft	0.82	0.21	2.28 (1.51–3.45)	< 0.001*
MM resection	− 0.29	0.13	0.75 (0.57–0.98)	0.03*
MM repair	− 0.45	0.22	0.63 (0.40–0.98)	0.04*
LM resection	0.05	0.12	1.06 (0.83–1.34)	ns
LM repair	− 0.30	0.24	0.73 (0.45–1.18)	ns
Cartilage injury	− 0.15	0.12	0.86 (0.67–1.10)	ns

*LSI* limb symmetry index, *ACL* anterior cruciate ligament, *ACLR* anterior cruciate ligament reconstruction, *HT* hamstring tendon, *MM* medial meniscus, *LM* lateral meniscus, *SE* standard error, *OR* odds ratio, *CI* confidence interval

*Statistically significant. *P* value < 0.05s

## Discussion

The most important finding of the present study was that only 19.6% of the patients achieved symmetrical knee function 6 months after ACLR, reaching a LSI of ≥ 90% in all three tests. The proportion of patients that achieved a LSI of ≥ 90% for isokinetic quadriceps muscle strength, hamstring muscles strength and the single-leg-hop test was 35.7%, 47.3% and 67.9%, respectively.

### Isokinetic quadriceps muscle strength

In the analysis of quadriceps muscle strength, age ≥ 30 years, female gender, MM repair and LM repair reduced the odds, whereas the use of HT autograft over BPTB autograft increased the odds of achieving symmetrical muscle strength. In a recent study, Ueda et al. [36] showed that older age and female gender are negatively associated with quadriceps strength recovery 6 months after ACLR. Iriuchishima et al. [12] also reported that older age is associated with residual muscle weakness 9 months after ACLR. These findings highlight that age- and gender-specific rehabilitation programs, with greater emphasis on quadriceps strength training for older and female patients, should be considered to facilitate strength recovery after ACLR.

Interestingly, patients who underwent a concomitant MM or LM repair had a lower odds of achieving symmetrical quadriceps muscle strength 6 months after ACLR. This is probably related to the fact that they underwent a different rehabilitation regime, with delayed onset of full weight-bearing and strength training due to the use of a postoperative hinged knee brace for the first 6 weeks after ACLR. As expected, graft choice between BPTB and HT autograft strongly affected the opportunity to achieve symmetrical quadriceps muscle strength. The use of HT autograft instead of BPTB autograft was found to be a factor strongly associated (OR, 3.41; 95% CI 2.48–4.69; *P* < 0.001) with the recovery of quadriceps muscle strength. Graft harvesting is accompanied by donor-site morbidity and muscle function impairment. A systematic review and meta-analysis [41] has shown that muscle weakness after ACLR is dependent on the graft donor site. Patients with HT autograft experience increased strength deficits with knee flexion, while patients with BPTB autograft experience increased strength deficits with knee extension.

### Isokinetic hamstring muscles strength

Age ≥ 30 years, delayed (> 3 months) ACLR, HT autograft and cartilage injury were found to be associated with a decreased odds of achieving symmetrical hamstring muscles strength, 6 months after ACLR. In a recent study, Krych et al. [16], reported that younger age and minimal cartilage degeneration are associated with excellent isokinetic muscle strength (LSI ≥ 85%) and functional hop performance (LSI ≥ 90%) 6 months after primary ACLR. Again, reduced muscle strength was found to be strongly correlated with the harvest site. For HT autograft ACLR, there has always been concern about hamstring weakness [15, 42]. It has been shown that HT autograft harvesting can reduce hamstring muscles strength for up to 1–2 years after ACLR [28]. Interestingly, time from injury to surgery longer than 3 months significantly reduced the odds of achieving symmetrical hamstring muscles strength. It can be hypothesized that, during the preoperative rehabilitation, more emphasis is generally placed on quadriceps muscle strengthening, resulting in a possible deconditioning of the hamstring muscles, in the event of a longer time interval from injury to surgery.

### Single-leg-hop test performance

Single-legged hop tests are performance-based measures used to assess the combination of muscle strength, neuromuscular control, confidence in the limb and the ability to tolerate loads related to sport-specific activities [20]. The single-leg-hop test is consistently reported in the literature to quantify knee performance after ACLR [10, 25, 40]. In the analysis of single-leg-hop test performance, age ≥ 30 years, female gender, delayed (> 3 months) ACLR, MM resection or MM repair and cartilage injury were found to be negatively associated with the opportunity to achieve a LSI of ≥ 90% six months after ACLR. Again, these findings highlight the fact that more attention should be paid to older and female patients. These categories of patients could need more specific rehabilitation programs after ACLR. As reported for the hamstring muscles strength analysis, a time from injury to surgery longer than 3 months was found to be associated with a lower opportunity to achieve a symmetrical single-leg-hop test performance. This could be due to the potential deconditioning of the injured knee over time. Patients with an ACL injury could experience a feeling of knee instability that could prevent their participation in physical activities, leading to the progressive deterioration of their knee function over time. Our study also revealed that the presence of MM resection, MM repair or cartilage injury was associated with a reduced odds of achieving limb symmetry for this test. This finding could be expected, as the presence of meniscal or cartilage injuries can cause knee pain [28], thereby resulting in a poorer single-leg-hop test performance. A pre-injury Tegner activity level ≥ 6 and the use of HT autograft over BPTB autograft were the two factors that increased the odds of achieving a symmetrical single-leg-hop test performance 6 months after ACLR. For the HT autograft ACLR, less anterior knee pain and kneeling pain than the BPTB autograft ACLR have been reported at a short-term follow-up [36]. These, together with the greater quadriceps strength associated with the use of this graft compared with BPTB autograft, could be responsible for the superior 6-month single-leg-hop test performance after ACLR performed with HT autograft. Patients with a high pre-injury Tegner activity level could begin rehabilitation with better knee function, be more eager to RTS, and therefore, spend more time and efforts in the rehabilitation process than patients with a low pre-injury activity level.

### Symmetrical knee function

When the effect of the independent variables on the achievement of a LSI ≥ 90% in all three tests (symmetrical knee function) was analyzed in our multivariate logistic regression analysis, it was found that age ≥ 30 years, MM resection and MM repair were the factors that reduced the odds, whereas the use of HT autograft over BPTB autograft was the only factor that increased the odds of achieving symmetrical knee function 6 months after ACLR.

It is accepted that muscle loss occurs with aging [30]. There is a link between age and loss of muscle fiber number and size [26]. However, the results of our study were expressed for both isokinetic muscle strength and single-leg-hop test performance as LSIs, comparing the ACL—reconstructed knee with the healthy knee. A muscle loss with aging could also be expected in the healthy knee, thereby not influencing the LSI. It is possible that, for older patients, the reduced odds of achieving symmetry for isokinetic muscle strength and single-leg-hop test performance could be related to a reduction in physical activities. Older patients could have lower knee demands compared with younger patients and they could, therefore, be less engaged in the rehabilitation process. The effect of a concomitant MM resection and MM repair on reducing the odds of achieving symmetrical isokinetic quadriceps muscle strength and single-leg-hop test performance has already been examined. These concurrent surgical procedures were still negatively associated with the opportunity to achieve a LSI of ≥ 90% in all three tests 6 months after ACLR, in our multivariate logistic regression analysis. The Graft choice between BPTB and HT autograft strongly influenced the likelihood of achieving symmetrical knee function six months after ACLR. A 2.28-fold (95% CI 1.51–3.45; *P* < 0.001) increase was found in the odds of achieving symmetrical knee function for patients receiving HT autograft. Although this finding could be interpreted as promoting the use of HT autograft over BPTB autograft for the earlier achievement of symmetrical knee function and RTS, it should be interpreted with caution. The ligamentization of the ACL graft occurs over a period of 1–2 years after surgery [5, 29] and the risk of sustaining an ACL re-injury is greatest during the early period (6–12 months) of RTS [8, 17]. Recent studies from the Scandinavian ACL registries [7, 23] reported an increased risk of revision with HT autograft compared with BPTB autograft.

The restoration of symmetrical quadriceps and hamstring muscles strength and single-leg-hop test performance is a crucial goal of rehabilitation after ACLR [8, 17, 34]. Muscular asymmetries are known to be a risk factor for ACL graft tears and knee re-injuries [8, 17]. Patients with a symmetrical isokinetic muscle strength and hop performance 6 months after ACLR have superior knee function and higher activity levels at the mid-term follow-up [31]. Quadriceps muscle strength is associated with patient-reported knee outcomes and satisfaction [13, 14, 19, 43], as well as with osteoarthritis development [22] after ACLR. Hamstring muscles strength deficits might be a risk factor for ACL re-tears, since they act as agonists to the ACL by resisting anterior tibial translation [11, 33].

The present study showed that the majority of patients are yet to regain symmetrical strength and function 6 months after ACLR. Increases in muscle volume and strength take a considerable length of time [39]. It is possible that, in most cases, 6 months are not sufficient to achieve symmetrical muscle strength and function after ACLR [34]. Even if recent studies suggested that RTS should not be permitted within 6 months after ACLR [8, 17], evaluating patients at this point provides clinicians with valuable information regarding patients’ current knee function, their necessity for additional rehabilitation and their readiness to RTS [20]. Patients with identified knee function asymmetries, 6 months after ACLR, have time and could benefit from targeted interventions before their transition to a more complex rehabilitation and their gradually RTS. Overall, the most common deficit among the tests was found for isokinetic quadriceps strength. These results are in line with those in other investigations that have looked at isokinetic strength after ACLR [38, 40]. Rehabilitation protocols should be implemented and more time needs to be spent on muscle strength rehabilitation, especially for the quadriceps muscle.

The main strength of this study is the large sample size (4093 patients). This allowed a robust logistic regression analysis and a detailed study of several factors affecting the achievement of symmetrical knee strength and function after ACLR, such as all types of meniscal surgery, that have not been investigated in previous studies. All patients received surgery and isokinetic muscle strength and single-leg-hop test performance assessment at the same institution. Moreover, the study cohort represented a wide range of patients regarding age, time from injury to surgery, pre-injury activity level and concomitant meniscal surgery. The results of this study are, therefore, highly generalizable.

There are several limitations. First, even if a standardized rehabilitation protocol has been followed after ACLR, the study timeframe is long. Some small changes in rehabilitation could, therefore, have occurred over time. Second, information regarding preoperative muscle strength and knee pain during testing was not available. It has been shown that these variables are independently associated with quadriceps muscle strength 6 months after ACLR [36]. Third, there was no information regarding other hop tests that have been reported in previous studies [8, 9, 16, 17, 20]. These tests were not routinely employed at our institution for all patients.

The results of the present study have significant implications for the clinical management of patients after ACLR. To our knowledge, this is the first large cohort study which clearly shows that most patients do not achieve symmetrical knee function 6 months after primary ACLR. In addition, it provides unique data, comprising a detailed analysis of several factors affecting the achievement of symmetrical isokinetic muscle strength and single-leg-hop test performance after ACLR, which have not been investigated in previous studies. Every effort should be made to correct knee function asymmetries between the ACL-reconstructed knee and the healthy knee and the results of this study should be taken into consideration when planning rehabilitation after ACLR. With knowledge of the factors affecting the achievement of symmetrical knee function, rehabilitation protocols should be developed to be more customized/targeted, depending on patient characteristics, and therefore, more effective for every single patient.

## Conclusion

Only 19.6% of the patients achieved symmetrical knee function 6 months after primary ACLR. Age ≥ 30 years, MM resection and MM repair reduced the chance, whereas the use of HT autograft over BPTB autograft increased the chance of achieving symmetrical knee function 6 months after primary ACLR. This study shows that most of the patients are yet to regain symmetrical knee function 6 months after primary ACLR and, moreover, it identifies several factors affecting its achievement in a large cohort. The results of this study should be used to counsel patients about their expected functional recovery and to optimize rehabilitation and maximize knee function after ACLR.
